# Extracellular proteins OSM-7 and OSM-8 are required for stress response gene regulation at all post-embryonic stages

**DOI:** 10.17912/micropub.biology.000688

**Published:** 2022-12-13

**Authors:** Victoria Rodriguez Mendoza, Luke Chandler, Zhexin Liu, Lauren Buddendorff, Amal Al-Rajhi, Thine Choi, Gabrielle Gibb, Justin Harvey, Alva Mihalik, Sophie Moravec, Wendy Pilcher, Vikram Raju, Keith Patrick Choe

**Affiliations:** 1 University of Florida, Gainesville, FL USA; 2 Department of Biology and Genetics Institute, University of Florida, Gainesville, FL USA

## Abstract

Nematode cuticles are extracellular matrices (ECMs) that function as structural support and permeability barriers. Genetic disruption of specific cuticle collagen structures or secreted epidermal proteins in *C. elegans* activates stress response genes in epithelial cells suggesting the presence of an extracellular damage signaling mechanism. Cuticles are replaced during development *via* molting but investigations of extracellular signaling to stress responses have focused on adults. In our current study, we measured cuticle phenotypes and stress response gene expression in all post-embryonic stages of mutant strains for a collagen and two secreted epidermal proteins to gain insights into developmental patterns.

**Figure 1. Cuticle phenotypes and stress response gene expression in dpy-5 , osm-7 , and osm-8 worms.**
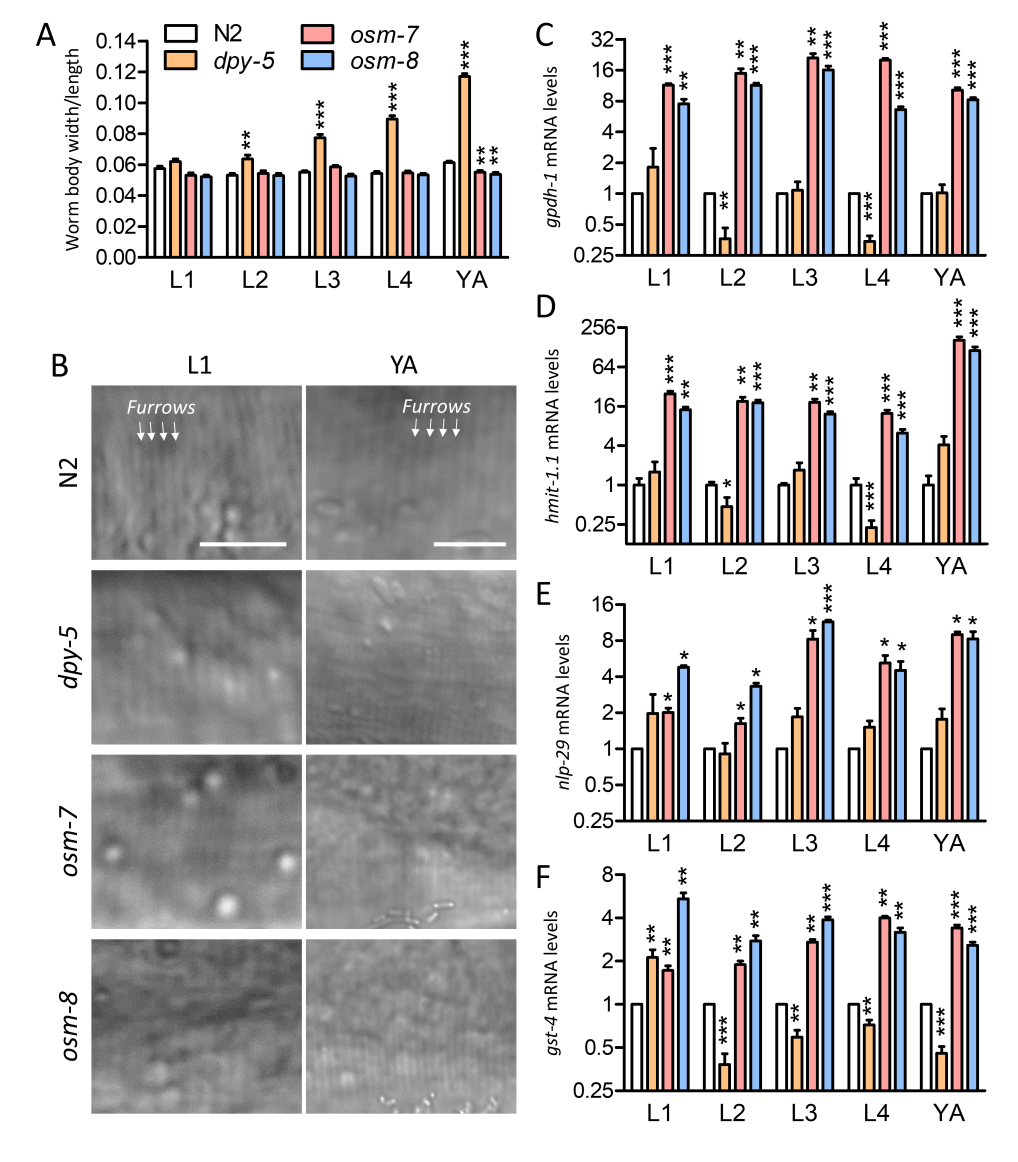
Mean plus standard error for worm body shape (A) and mRNA levels of representative stress response genes (C-F). *P<0.05, **P<0.01, and ***P<0.001; N = 10-17 individual worms in A and 4-6 replicate populations of worms in C-D. Representative high magnification DIC images of the cuticle are shown in B and were similar in all stages and individual worms. Scale bars are 5 μm in B.

## Description

Extracellular matrices (ECMs) act as barriers to environmental stress on the outer surface of animals. Within cells, well-studied molecular pathways sense stress and regulate cytoprotective gene expression (Choe et al. 2009, Blackwell et al. 2015, Urso and Lamitina 2021, Pujol and Ewbank 2022). Despite being in direct contact with the environment, little is known about the role of barrier ECMs in sensing environmental conditions and regulating stress response genes in underlying cells.

Nematodes have a tough and flexible collagen-based barrier ECM called the cuticle that is re-synthesized and molted during transitions between post-embryonic developmental stages (Cox and Hirsh 1985, Johnstone 2000, Page and Johnstone 2007). We and others discovered that genetic disruption of collagens that form repeating ‘annular furrow’ structures in the superficial layer of the adult cuticle activates osmotic, detoxification, and innate immune response genes in epithelial cells (Lamitina et al. 2006, Pujol et al. 2008, Dodd et al. 2018). Manipulation of other cuticle and epidermal structures does not have the same effects on these stress responses and heat shock and organelle-specific stress responses are not activated by furrow disruption (Dodd et al. 2018). These results are consistent with a damage signaling pathway in adults that is associated with furrows. There are six collagens that are required for organization of furrows in adults and they belong to a larger class of 26 *dpy* genes that cause a short and wide body shape when mutated or silenced (McMahon et al. 2003).

All post-embryonic stage cuticles have furrows, but other cuticle structures, collagen gene expression, and thickness vary between stages (Cox et al. 1981, Hendriks et al. 2014). We recently demonstrated that furrow collagen gene mutations cause a progressive loss of furrow organization and body shape starting at the L3 stage (Chandler and Choe 2022); among stages, stress response gene expression was induced the strongest in adults correlated with the strongest furrow disorganization and Dpy phenotypes.

To gain further insights into extracellular stress response signaling during development, we investigated worms with mutations in a *dpy* collagen gene not required for furrow organization in adults *(dpy-5)* and non-collagen secreted protein genes *osm-7* and *osm-8* that are required for regulation of the same stress responses as furrow collagens without any obvious influence on body shape (Wheeler and Thomas 2006, Rohlfing et al. 2010, Wimberly and Choe 2022). Adult *dpy-5* loss-of-function worms have a strong Dpy body shape phenotype but have organized furrows and do not activate stress responses (Dodd et al. 2018). Adults of *osm-8* mutant worms were shown previously to have organized furrows (Rohlfing et al. 2011).

As shown in Fig. 1A, *dpy-5(e61)* worms developed a Dpy phenotype starting at the L2 stage that became progressively stronger with each molt. The Dpy phenotype has a similar developmental pattern in furrow collagen gene mutants albeit weaker and starting later than in *dpy-5(e61)* worms (Chandler and Choe 2022). These progressive body shape phenotypes are consistent with synthesis of each new cuticle being at least partially reliant on organization of the former (Chandler and Choe 2022).

Representative high magnification DIC micrographs for L1 and young adult worm cuticles are in Fig. 1B; we observed organized furrows in all individuals and stages of *dpy-5* worms. We observed no Dpy phenotype for any stage of *osm-7(tm2256)* and *osm-8(n1518)* worms and slightly lower width/length ratios than wild type in adults (Fig. 1A). These worms had organized furrows at all stages (L1 and adult shown in Figs. 1B) consistent with OSM-7 and OSM-8 functioning outside or downstream of the cuticle (Wheeler and Thomas 2006).

RT-qPCR of representative canonical osmotic ( *gpdh-1* and *hmit-1.1* ), detoxification ( *gst-4* ), and innate immune response genes ( *nlp-29* ) are shown in Figures 1C-F; these genes are strongly induced in late larval and adult furrow collagen gene mutants and during exposure to hypertonicity (Dodd et al. 2018, Chandler and Choe 2022, Wimberly and Choe 2022). In *dpy-5* worms, expression of these stress response genes was either not different or reduced relative to N2 at all stages except for *gst-4* in L1 larvae, which was increased 2.1-fold. This lack of broad stress response gene activation indicates that a wide body shape is not sufficient to activate stress responses at any developmental stage.

In *osm-7* and *osm-8* worms, all four representative stress response genes were induced in all stages (Figs. 1C-F). There was no obvious developmental pattern in *gpdh-1* induction in either *osm-7* or *osm-8* worms; *hmit-1.1* was induced the greatest in adults (Figs. 1C-D). In *osm-7* worms, *nlp-29* and *gst-4* had a trend of greater induction in late stages, but there was no obvious pattern in *osm-8* worms (Figs. 1E-F). These data are consistent with the pathways responding to *osm-7* and *osm-8* loss functioning at all post-embryonic stages.

## Methods

Conceptualization, design of methodology, and preliminary assays were completed in an immersive CURE course (course-based undergraduate research experience) named “Molecular and Genetic Responses to Environmental Stress” at the University of Florida (Auchincloss et al. 2014, Wang 2017). Students from this course completed gene expression measurements and helped with body shape measurements.

Body shape was determined from images taken with a Zeiss Discovery V12 Stereo microscope and OptixCam Summit camera as a ratio of average width to total length using ImageJ version 1.53r as described previously (Schindelin et al. 2012, Chandler and Choe 2022). The superficial cuticle was imaged by DIC on an Olympus BX60 microscope with an oil 60× UPlanFL objective (numerical aperture at 1.25) and additional 2× lens in front of a Zeiss Axiocam MRm camera (Thornwood, NY) as described previously (Chandler and Choe 2022).

qRT-PCR assays were run using the delta-delta Ct method with primer efficiencies determined from standard curves as described previously (Scolaro et al. 2019, Chandler and Choe 2022) with the following modifications; after lysis, genomic DNA was removed with dsDNAse (Thermo Fisher product EN007). Numbers of worms in each replicate were adjusted to account for worm sizes as follows: 200 L1, 180 L2, 50 L3, and 15 L4 larvae for all strains; for young adults: 10 N2, *osm-7* , and *osm-8* and 12 *dpy-5* . All RT-qPCR reactions were performed in 10 μL volumes in a Realplex2 (Eppendorf AG, Hamburg, Germany). Relative expression was normalized to a value of 1.0 for N2 within each developmental stage using the average of three reference genes ( *rpl-2* , *tba-1* , and *cdc-42* ).

Body shapes for all mutant strains were compared to N2 with unpaired *t* -tests with Welch’s corrections (Ruxton 2006). All relative mRNA values were normalized within each stage to a value of 1.0 for N2 and compared to 1.0 with a one-sample t-test. P-values were false-discovery corrected with Benjamini-Hochberg adjustments. Numerical data underlying Figure 1 are in the Extended Data section.

## Reagents

*C. elegans* strains:

N2, CB61 *dpy-5(e61)* , HA1857 *osm-7(tm2256)* , and MT3571 *osm-8(n1518).* Strains were provided by the CGC, which is funded by NIH Office of Research Infrastructure Programs (P40 OD010440).

Primer sequences (5’ to 3’):

*rpl-2* F – CTTTCCGCGACCCATACAA

*rpl-2* R – CACGATGTTTCCGATTTGGAT

*hmit-1.1* qF1 - TAGTGTCGGCTGCAATGCTTT

*hmit-1.1* qR1 - CGCCGACCAATATAGTCGGA

*gpdh-1* qPCR F - TTATGAGGCCGTGGAGCTTT

*gpdh-1* qPCR R - CCAGACGGATGATAGCGGAT

*nlp-29* F - CGAGGAATGTATGGAGGCTATG

*nlp-29* R - TCCATGTATTTACTTTCCCCATCC

*cdc-42* F3 - CGTTGACGCAGAAGGGACT

*cdc-42* R3 - GAGAAGAGTGGAAGTCGGGG

*tba-1* F - TCAACACTGCCATCGCCGCC

*tba-1* R - TCCAAGCGAGACCAGGCTTCAG

## Extended Data

Description: Extended Data - Numerical data underlying Figure 1. Resource Type: Dataset. DOI: 10.22002/d1969-eec25
